# Efficacy and safety of herbal medicine (Bailemian capsule) for treating insomnia: Protocol for a systematic review and meta-analysis

**DOI:** 10.1097/MD.0000000000014275

**Published:** 2019-01-25

**Authors:** Tian Tian, Lian Hua, Jianxin Wang, Jingzhi Guan

**Affiliations:** a Department of Neurology, Guizhou Provincial People's Hospital, Guiyang, Guizhou; b College of Mongolian Medicine, Inner Mongilia Medical University, Hohhot, Inner Mongolia; c Human Anatomy and Tissue Embryology, Weifang Medical University, Weicheng, Weifang, Shandong; d Department of Pharmacy, Inner Mongolia International Mongolian Hospital, Hohhot, Inner Mongolia, China.

**Keywords:** Bailemian capsule, herbal medicine, insomnia, meta-analysis, protocol for a systematic review

## Abstract

**Background:**

Insomnia has become a universal subhealth disease, affecting more and more modern people's health and quality of life. At present, western medicine is only symptomatic treatment for insomnia. Bailemian Capsule (BLMC) is a proprietary Chinese medicine for treating insomnia. It has been widely used in China, but lacks evidence for evidence-based medicine. At the same time, the clinical efficacy and safety of BLMC are controversial. Therefore, the effectiveness and safety of BLMC in the treatment of insomnia are studied and systematically evaluated in this study. It provides reliable theoretical support for the treatment of insomnia with Traditional Chinese Medicine and the combination of traditional Chinese and Western medicine.

**Methods:**

The information was retrieved from electronic databases, Cochrane, PubMed, EMBASE, SinoMed, China National Knowledge Infrastructure, VIP Data, and WangFang Data. Randomized controlled trials on the BLMC in the treatment of insomnia were conducted. There was no limitation on the literature language. RevMan 5.3 software and STATA 12.0 software were used to perform the meta-analysis.

**Results:**

This review will be to assess the efficacy and safety of BLMC for insomnia.

**Conclusion:**

Our systematic evaluation will provide evidence for the clinical efficacy and safety of BLMC in the treatment of insomnia, and will be published in the form of academic papers in the future to provide new ideas for clinicians in the treatment of insomnia.

## Abstract 2

Supplemental Digital Content is available in the text

## 1 Introduction

### 1.1 Current status of insomnia

Insomnia refers to a lack of normal sleep time due to various reasons. Physiologically, insomnia reflects the weakening of the suppression function. Clinically, insomnia is characterized by difficulty in falling asleep and a sleeping time of no more than 30 minutes; the quality of sleep is decreased, sleep is maintained, and the number of awakenings is ≥2, with early waking and declined sleep quality; total sleep time (TST) is reduced and is usually <6 hours with nightmares and dreams.^[1]^ With the increasingly fierce competition and the accelerated pace of life, many of us suffer insomnia to different degrees. If people are so anxious about the initial insomnia symptoms that they cannot remove the interference factors,^[2]^ as these symptoms develop with time, then the initial symptoms tend to be fixed and eventually develop into insomnia. Reports have shown that among all complaints about sleep, insomnia is the most common. A 1979 Gallup poll shows that 95% of American adults suffer from insomnia.^[3]^ Based on this Gallup poll, half of the adults in the United States suffer from sleep disorders; 35% are reported to have insomnia, and 12% are reported to have serious insomnia. In the United States, 300 hundred million to 1080 hundred million dollars are used for the treatment of sleep disorders each year, leading to a great consumption of medical resources, a decline in individual social function and productivity, an increase in the number of sick leave days, and thus a great influence on the overall economic power of the whole country.^[4]^ In the current society, the incidence of insomnia among Chinese adults can reach 5% to 45%, and insomnia is more common among elderly individuals. Among several types of sleep disorders, the incidence of insomnia is the highest, accounting for approximately 97.5%.^[5]^

### 1.2 Description of the intervention

Currently, western medicine treats insomnia by drug therapy, including first-generation barbiturates, second-generation benzodiazepines, and third-generation nonbenzodiazepines. First-generation barbiturates are rarely used in the clinic. Second-generation benzodiazepines are the most widely used medicine in the treatment of insomnia, and they can shorten the time for falling asleep, reduce the awakening time and frequency, and increase total hours of sleep. However, this kind of medicine also has disadvantages because it is easy to form drug dependence and experience discontinuation of recoil and memory deterioration.^[6]^ From ancient times, Traditional Chinese Medicine (TCM) has recorded insomnia as “bumei” (sleepless in Chinese). Based on the theory of TCM, insomnia is caused by many factors, including the enfeeblement of viscera and the imbalance of Yin and Yang, which lead to interference of the mind. In contrast to western medicine, which treats insomnia at the surface, TCM focuses on developing the general symptoms to treat both the manifestation and root cause of insomnia. As a Chinese patent medicine against insomnia, the Bailemian capsule (BLMC) in this article is made of 15 Chinese medicines: Mother-of-pearl; *Lilium brownii* F.E. Brown ex Miellez var. viridulum Baker; Gypsum; Spina Date Seed; *Albizia julibrissin* Durazz; Tuber Fleeceflower Stem; *Codonopsis pilosula* (Franch.) Nannf; *Rehmannia glutinosa* (Gaertn.) Li-bosch; *Polygala tenuifolia* Willd; *Schisandra chinensis* (Turcz.) Baill; *Juncus effusus* Linn; *Acanthopanax senticosus* (Rupr. et Maxim.) Harms; *Ophiopogon japonicus* (Linn. f.) Ker-Gawl; *Salvia miltiorrhiza* Bunge; and *Smilax glabra* Roxb, which has been widely used in China.^[7]^ Some studies have shown that the BLMC has a good curative effect in treating insomnia. Therefore, based on the theory of TCM and evidence-based medicine, this article collected and systematically evaluated randomized controlled trials (RCTs) of the BLMC in the treatment of insomnia that were completed or published before May 1, 2018. To this end, the aims of this study were to systematically study and evaluate the effectiveness and safety of the BLMC in the treatment of insomnia based on the available literature and to provide reliable theoretic support for the treatment of insomnia by TCM and by the combination of western medicine and TCM. This study has been supported by the Fund. For relevant information, see Supplemental.

## 2 Methods

### 2.1 Protocol and registration

A protocol had been registered for this systematic review and meta-analysis in PROSPERO (CRD42018114512) (https://www.crd.york.ac.uk/PROSPERO).

### 2.2 Ethics and dissemination

This proposed meta-analysis does not require ethical approval and informed consent of patients, because the system evaluation does not involve patient recruitment, and the data used in this study are from published research papers. The results of this study will be published in peer-reviewed international journals.

### 2.3 Eligibility criteria

#### 2.3.1 Types of studies

RCTs about the BLMC in the treatment of insomnia were retrieved, regardless of whether a blinding method (single blind, double blind, or triple blind) was adopted in the selected study. This study is based on the preferred reporting items for systematic reviews and meta-analysis (PRISMA statement).

#### 2.3.2 Types of participants

The selected patients should be diagnosed with primary or secondary insomnia. There should be clear diagnostic criteria (Chinese medicine diagnostic criteria or Western diagnostic criteria are not limited), no other drugs had been taken before the study. Patient's age, gender, time of illness, and whether other diseases are not restricted.

#### 2.3.3 Ethics approval

This study is a systematic review. All patient data are obtained from published papers.

#### 2.3.4 Types of interventions

The experimental group received single therapy with the BLMC alone or in combination with western medicine or TCM (no limitation on the dose, administration frequency, course of the disease, primary insomnia or secondary insomnia, administration interval or course of treatment). The control group received single therapy consisting of western medicine or TCM (no limitation on the dose, administration frequency, course of the disease, primary insomnia or secondary insomnia, administration interval or course of treatment).

#### 2.3.5 Types of outcome measures

##### 2.3.5.1 Primary outcomes

The total effective rate was calculated based on the criteria for the therapeutic effects of western medicine and TCM. Refer to the criteria for the efficacy of insomnia in Guiding Principles for Clinical Research of New Chinese Medicine.^[8]^ Clinical recovery: sleep time returned to normal or increased to >6 hours per night, deep sleep, wake up energetic. Markedly effective: sleep time increased by >3 hours, and sleep depth increased. Effective: sleep time increased by <3 hours, and symptoms relieved. Invalid: no improvement or aggravation of insomnia; TCM syndrome curative effects standard: it is divided into 4 grades: those whose symptoms disappear after treatment are cured; those whose integral value decreases 2/3 after treatment are effective; those whose integral value decreases 2/3 to 1/3 after treatment are effective; those whose integral value decreases <1/3 after treatment are ineffective. The total effective rate = (Clinical recovery + Markedly effective + Effective)/Total number of cases × 100%.

##### 2.3.5.2 Secondary outcomes

Pittsburgh Sleep Quality Index score^[9]^: it is widely used in clinical evaluation of insomnia. It is characterized by the organic combination of the quality and quantity of sleep to assess, indicators include patients’ sleep quality, sleep time, sleep efficiency, sleep disorders, hypnotics, and daytime functions. A total of 4 surveys were conducted for each patient (before treatment, l, 2, 3 weeks after treatment).

Recurrence rate.

Adverse reaction ratio.

### 2.4 Search methods for the identification of studies

#### 2.4.1 Electronic searches

The network electronic databases were searched using a computer. The retrieved foreign databases included the Cochrane Library, PubMed, and EMBASE, while the retrieved Chinese databases included SinoMed, China National Knowledge Infrastructure (CNKI), the Chinese Biomedical Literature Database (VIP) Data, and WangFang Data. A combination of keywords and free words was used as the retrieval strategy.

#### 2.4.2 Other sources

Manual search of supplements to the following magazines (no electronic version of the file), including “China Journal of Chinese Materia Medica,” “Chinese Journal of Experimental Traditional Medical Formulae,” “China Journal of Chinese Traditional Medicine and Pharmacy,” “Shizhen Journal of Traditional Chinese Medicine,” and “Journal of Traditional Chinese Medicine,” related conference papers and papers collected after 2010 were retrieved by hand.

#### 2.4.3 Search strategy

The network electronic databases were searched using a computer. The retrieved foreign databases included the Cochrane Library, PubMed, and EMBASE, while the retrieved Chinese databases included SinoMed, CNKI, VIP Data, and WangFang Data. A combination of keywords and free words was used as the retrieval strategy. Chinese retrieval words included “Bailemian,” “Shimian,” “Bumei,” and “Shuimianzhangai,” while English retrieval words included “Bailemian capsule,” “Bailemian Capsule,” “insomnia,” “agrypnia,” “hyposomnia,” “sleeplessness,” “dyssomnia,” “sleep disorders,” and “sleep disorder.” There was no limitation on the language. The literature search strategies used are shown in Table 1.

**Table 1:**
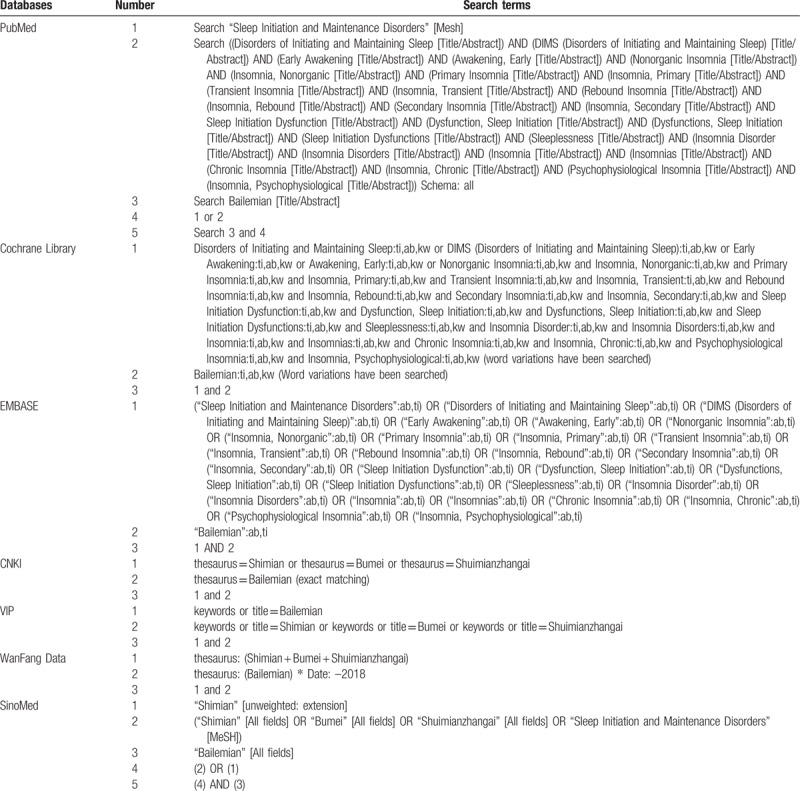
Search strategy for the network electronic database.

### 2.5 Data collection and analysis

#### 2.5.1 Data extraction

**Figure 1:**
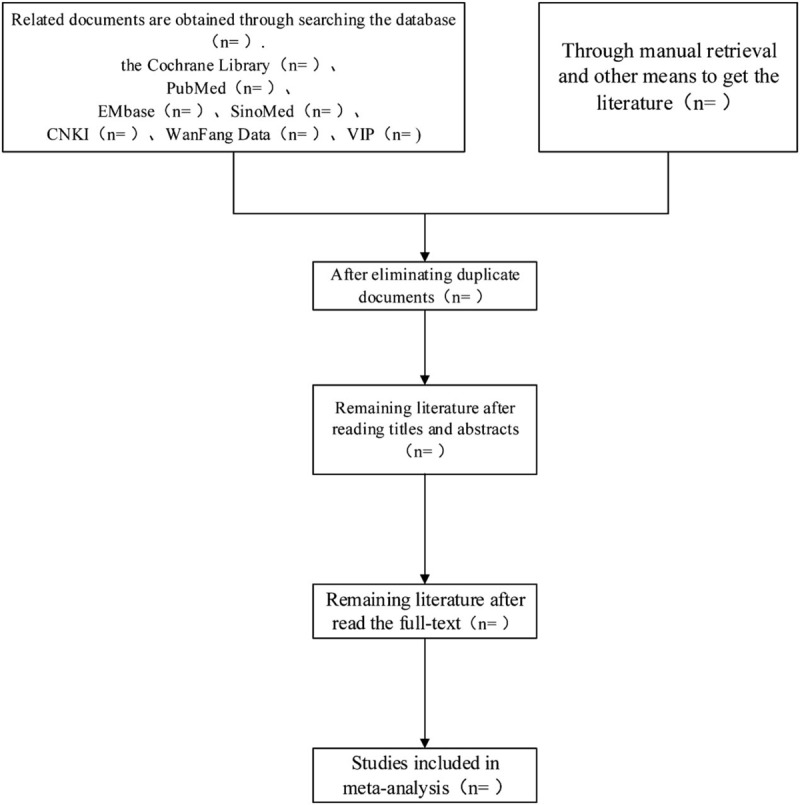
Flow chart of study selection.

Two researchers (Jianxin Wang and Lian Hua) separately read the titles and abstracts of the retrieved literature and excluded those that did not meet the standards. For the remaining literature that met the absorption standards, the researchers obtained the full texts for further exclusion, and a screening table was created to indicate the reasons for exclusion. Finally, the researchers conducted data extraction of the absorbed literature, including author name, publication time, gender ratio, average age, average course of the disease, number of positive cases in the experimental and control groups, course of treatment, diagnosis standard, curative effect standard, and final index. Two researchers crosschecked the average results and determined whether to receive the controversial literature by discussion or invite a third reviewer who had rich experience in meta-analysis to arbitrate. The screening process of the paper is shown in Fig. 1.

#### 2.5.2 Addressing missing data or unclear measurement scales

For papers with incomplete data coverage, the author of the paper is first requested by e-mail or telephone. If the author of the paper does not agree to provide data or cannot contact the author of the paper, we will discuss the trade-offs of the paper.

#### 2.5.3 Assessment of heterogeneity

Based on risk of bias assessment tool, the quality evaluation included the following 6 aspects^[10]^: random allocation; allocation concealment; the use of an objective blind method, treatment options, and results measurement personnel; data integrity; selective results reporting; and other sources of bias (publication bias, language bias, geographical bias, etc.). Two evaluators performed quality evaluations by reading the full text separately. Then, the quality evaluation results were crosschecked. Controversial results were managed by discussion or evaluation by a third party.

#### 2.5.4 Data analysis

Meta-analysis was conducted by RevMan 5.3 software. In this article, the risk ratios and 95% confidence interval (CI) were used for the effect analysis of statistics to evaluate the ending calculation data. For the same unit, the weighted mean differences and the 95% CI analysis were used for the continuous data, and for different units, the standardized mean difference and 95% CI were used. The heterogeneity of the research results was analyzed by a chi-squared test (with the test level, α = 0.1), and the size of the heterogeneity was determined by combining with the I^2^ quantification. If there was statistical homogeneity (*P* > .1, I^2^ ≤ 50%) among the research results, then the fixed-effect model was used for the meta-analysis. If there was statistical heterogeneity (*P* ≤ .1, I^2^ > 50%) among the research results, a random-effect model was used for the meta-analysis after excluding the influence of obvious clinical heterogeneity. Obvious clinical heterogeneity was managed by subgroup analysis, sensitivity analysis, or descriptive analysis.

#### 2.5.5 Sensitivity analysis

To test the certainty of the results, we carried out a sensitivity analysis, removing studies that would have a greater impact on the results if the results remained unchanged and would ascertain their certainty. On the other hand, if the results changed, then we would have to analyze and examine the removed studies more carefully to find the source of heterogeneity and draw a cautious conclusion or carry out a descriptive analysis of the results.

#### 2.5.6 Subgroup analysis and solutions to heterogeneity

For outcome indicators with more heterogeneous results, we carried out subgroup analysis on the more heterogeneous results, based on information found in the literature, to determine the reason for the increased heterogeneity. Simultaneously, with appropriate subgroup analysis, we can reduce the degree of heterogeneity and increase the certainty of the results. In this study, subgroup analysis was conducted according to the intervention measures, which had a great influence on outcome indicators, and was divided into BLMC versus western medicine and BLMC versus other Chinese medicine.

#### 2.5.7 Assessment of reporting bias

For the heterogeneous results, publication bias was tested using Egger test in STATA 12.0 software, and *P* < .05 concluded that there was publication bias in outcome indicators.

## 3 Discussion

Insomnia is a common disease with symptoms including difficulty falling asleep and early awakening. This condition features a reduction in the time and depth of sleeping. Currently, with the increasingly faster pace of life, the incidence of insomnia is also increasing. This increase may lead to great harm, such as lower productivity and psychological barriers. Serious insomnia may cause certain physiological and psychological diseases, including mood disorders, a rise in blood pressure or blood sugar, anorexia, and even suicide due to depression.^[11]^ The combination of western medicine and TCM can overcome this slow effect and improve symptoms in a short time.^[12]^ In this way, patients will show higher dependence and continue the therapy.

Moreover, the dosage of western medicine will be reduced, which can also prevent the side effects caused by single western medicine therapy. Current studies have shown that BLMC is a safe and effective drug for treating insomnia. First, *L brownii* F.E. Brown ex Miellez var. viridulum Baker contains lily saponins, which can significantly reduce the independent activity and prolong the sleep time of mice caused by pentobarbital sodium, and the sedative and hypnotic effects of these saponins increase with increasing dose^[13]^; *S chinensis* (Turcz.) Baill and *A senticosus* (Rupr. et Maxim.) Harms have inhibitory effects on the central nervous system. Li et al studied the effect of *A senticosus* saponin extract on sleep in mice through animal experiments. The sleep latency, incidence, and prolonged time in the *A senticosus* saponin group and control group were observed within 30 minutes after pentobarbital injection. The results showed that *A senticosus* saponin extract can shorten sleep latency, increase the incidence of sleep, and prolong the sleep time in mice. In addition, *A senticosus* saponins have no direct effect on the sleep of the mice, suggesting that *A senticosus* can promote sleep in mice.^[14]^*Schisandra chinensis* (Turcz.) Baill is a raw material that can be used in health food. Yang observed the effect of *S chinensis* (Turcz.) Baill decoction on the sleep phase of free-moving rats by animal experiments, and the results showed that *S chinensis* (Turcz.) Baill decoction could prolong the TST of normal rats. In the sleep time phase, slow wave sleep phase II was significantly prolonged during sleep, but slow wave sleep phase I and fast wave sleep were not affected. These results showed that the decoction of *S chinensis* (Turcz.) Baill has the effect of improving sleep^[15]^; *A senticosus* (Rupr. et Maxim.) Harms is good for clearing heat and relieving restlessness. *Smilax glabra* Roxb is good for relieving the uneasiness of the mind and nourishing the blood. The combination of the above medicines functions to significantly relieve the uneasiness of the mind, nourish the blood, relieve restlessness, and ease heart palpitations.

Through the systematic evaluation of the effect of the BLMC on the treatment of insomnia, this article aimed to increase the sample size, improve the reliability, and provide reliable theoretical support for clinical treatment based on meta-analysis.

## Author contributions

This work was done by the authors named in this article. Jingzhi Guan and Tian Tian designed the study and wrote the article. Jingzhi Guan, Tian Tian, Lian Hua, and Jianxin Wang collected and analyzed the data. Jingzhi Guan reviewed the full text and proofread the manuscript.

**Conceptualization:** Tian Tian, Jingzhi Guan.

**Data curation:** Tian Tian, Lian Hua, Jianxin Wang, Jingzhi Guan.

**Investigation:** Jingzhi Guan.

**Resources:** Lian Hua, Jianxin Wang.

**Software:** Tian Tian.

**Writing – original draft:** Tian Tian, Lian Hua, Jianxin Wang.

**Writing – review & editing:** Jingzhi Guan.

Jingzhi Guan orcid: 0000-0002-3086-477X.

## Supplementary Material

Supplemental Digital Content

## Supplementary Material

Supplemental Digital Content
