# Supplementary material for: Efficacy and safety of herbal medicine (Bailemian capsule) for treating insomnia: Protocol for a systematic review and meta-analysis
Source: Medicine (Baltimore). 2019 Jan 25;98(4):e14275. doi: 10.1097/MD.0000000000014275 (PMC6358357; doi:10.1097/MD.0000000000014275)
Supplement: Supplemental Digital Content [file medi-98-e14275-s001.pdf]

内蒙古自治区卫生和计划生育委员会

内卫计科教字〔2017〕740号

内蒙古自治区卫生计生委关于公布2017年度自治区卫生计生科研计划项目的通知

各盟市卫生计生委，满洲里市、二连浩特市卫生计生局，委直各单位，自治区各高等医学院校及其直属附属医院，各有关医疗卫生计生单位：

为了进一步落实科教兴医战略，推进卫生计生科技创新，自治区卫生计生委于 2017 年组织各级各类卫生计生机构申报科研计划项目，得到全区卫生计生科技工作者的积极响应。经自治区卫生计生委组织相关专业的资深专家严格评审，确定了 2017 年自治区卫生计生委卫生计生科研计划项目 562 项，其中，A 类一等课题 120 项，A 类二等课题 180 项，B 类课题 262 项，现予公布。请各地各单位按照项目内容，认真落实项目任务。现将有关事宜通知如下：

一、2017 年自治区卫生计生委卫生计生科研计划项目课题补

助为 A 类一等 2 万元，A 类二等 1 万元。A 类一等、A 类二等课题分别由自治区卫生计生委 2017—2019 年科研教育经费预算拨付，B 类课题为单位和课题负责人自筹资金课题。各地各单位可根据实际情况，对自治区卫生计生委给予的补助经费予以配套（原则上不低于 1:1 的比例），同时对 B 类自筹资金课题予以支持，确保科研计划项目经费的需要，自治区卫生计生委将不定期对科研补助经费使用及科研项目工作开展情况进行抽查。

二、请列入计划项目的负责人，按照要求认真填写《2017 年内蒙古自治区卫生和计划生育委员会医疗卫生科研计划项目任务书》（一式五份），加盖单位公章后，于 2017 年 12 月 20 日前由盟市卫生计生委统一报至自治区卫生计生委，自治区直属单位可直接报至自治区卫生计生委。

联 系 人：自治区卫生计生委科教处　袁慧忠

联系电话：6946202

地　　址：呼和浩特市新华大街 63 号 8 号楼

邮　　编：010055

附件：1.2017年度自治区卫生计生科研计划项目立项名单

2.2017年度自治区卫生计生科研计划项目任务书

内蒙古自治区卫生计生委

2017 年 11 月 27 日

（信息公开形式：主动公开）

发

内蒙古自治区卫生计生委办公室　　　　　　　2017年11月27日印

发

校对：袁慧忠 共印 50 份
