# Supplementary material for: Efficacy and safety of herbal medicine (Bailemian capsule) for treating insomnia: Protocol for a systematic review and meta-analysis
Source: Medicine (Baltimore). 2019 Jan 25;98(4):e14275. doi: 10.1097/MD.0000000000014275 (PMC6358357; doi:10.1097/MD.0000000000014275)
Supplement: Supplemental Digital Content [file medi-98-e14275-s002.pdf]

项目编号	项 目 名 称	申 请 单 位	课题负责人	立项类别	拨款年度	万元
**201703037**	骨盆骶骨固定术在骶骨肿瘤切除重建中的应用	内蒙古自治区国际蒙医医院	段新民	**B**类	自筹	
**201703038**	化浊益肾**7**味丸为主的蒙药治疗慢性肾炎的临床疗效研究	内蒙古自治区国际蒙医医院	宝玉	**B**类	自筹	
**201703039**	基于古籍文献的蒙医妇科诊疗理论研究	内蒙古自治区国际蒙医医院	张玉荣	**B**类	自筹	
**201703040**	关于蒙医基础“五元学”理论的科学依据研究	内蒙古自治区国际蒙医医院	张全	**B**类	自筹	
**201703041**	蒙药与西药治疗原发免疫性血小板减少症的药物经济学比较	内蒙古自治区国际蒙医医院	宝山	**B**类	自筹	
**201703042**	中药百乐眠胶囊治疗失眠症临床随机对照试验的**Meta**分析	内蒙古自治区国际蒙医医院	关敬之	**B**类	自筹	
**201703043**	分子影像**MicroPET/CT**评价蒙药广枣总黄酮对心肌缺血/再灌注损伤的保护作用	内蒙古自治区国际蒙医医院	韦丽虹	**B**类	自筹	
**201703044**	蒙古族与汉族患者疼痛敏感性及舒芬尼术后镇痛效果对比性研究	内蒙古自治区国际蒙医医院	张远	**B**类	自筹	
**201703045**	蒙医心身互动疗法治疗溃疡性结肠炎的临床研究	内蒙古自治区国际蒙医医院	白玉凤	**B**类	自筹	
**201703046**	胃肠超声造影诊断胆汁反流性胃炎经蒙药治疗效果评估	内蒙古自治区国际蒙医医院	王俊娥	**B**类	自筹	
**201703047**	基于蒙古族与汉族特性的免疫细胞亚群在冠心病发生发展过程中的临床应用研究	内蒙古自治区国际蒙医医院	王青春	**B**类	自筹	
**201703048**	石膏、夹板固定胫腓骨骨折	内蒙古自治区国际蒙医医院	杜孟特	**B**类	自筹	
**201703049**	蒙药治疗重症布病优势分析	内蒙古自治区国际蒙医医院	德胜	**B**类	自筹	
